# Are non-high–density lipoprotein fractions associated with pediatric metabolic syndrome? The CASPIAN-V study

**DOI:** 10.1186/s12944-018-0895-1

**Published:** 2018-11-14

**Authors:** Pooneh Angoorani, Majid Khademian, Hanieh-Sadat Ejtahed, Ramin Heshmat, Mohammad Esmaeil Motlagh, Mahya Vafaeenia, Gita Shafiee, Armita Mahdivi-Gorabi, Mostafa Qorbani, Roya Kelishadi

**Affiliations:** 1 Chronic Diseases Research Center, Endocrinology and Metabolism Population Sciences Institute, Tehran University of Medical Sciences, Tehran, Iran; 2 Department of Pediatrics, Child Growth and Development Research Center, Research Institute for Primordial Prevention of Non-communicable Disease, Isfahan University of Medical Sciences, Hezar-Jarib Ave, Isfahan, Iran; 3 Obesity and Eating Habits Research Center, Endocrinology and Metabolism Clinical Sciences Institute, Tehran University of Medical Sciences, Tehran, Iran; 4 Department of Pediatrics, Ahvaz Jundishapur University of Medical Sciences, Ahvaz, Iran; 5 Non-communicable Diseases Research Center, Alborz University of Medical Sciences, Karaj, Iran; 6 Endocrinology and Metabolism Research Center, Endocrinology and Metabolism Clinical Sciences Institute, Tehran University of Medical Sciences, Tehran, Iran

**Keywords:** Metabolic syndrome, Non-high-density lipoprotein cholesterol, Children, Adolescents

## Abstract

**Background:**

Non-high-density lipoprotein cholesterol (non-HDL-C) is considered as a valuable predictor for dyslipidemia and subclinical atherosclerosis which can be an appropriate index for identifying individuals with metabolic syndrome (MetS).

**Objective:**

To evaluate the association between non-HDL-C MetS and determine the optimal cut-points of non-HDL-C fractions for identifying MetS in Iranian children and adolescents.

**Methods:**

This nationwide study was conducted in the framework of the fifth survey of a national school-based surveillance program on children and adolescents aged 7–18 years. MetS was defined by the Adult Treatment Panel III (ATP III) criteria modified for the pediatric age group. The analysis of receiver operating characteristic (ROC) curve was applied to determine the optimal cut-points of non-HDL-C, difference between non-HDL-C and LDL-C (Diff-C) and triglycerides (TG) to HDL-C ratio (TG/HDL-C) for the prediction of MetS.

**Results:**

Overall, the study participants consisted of 3843 students (52.3% boys) with mean (±SD) age of 12.28 (3.1) years. The odds of high LDL-C, low HDL-C and MetS were increased in subjects with higher non-HDL-C, Diff-C and TG/HDL-C (*P* < 0.05). Non-HDL-C, Diff-C and TG/HDL-C cut-off points for predicting MetS were 120.5 mg/dl (sensitivity: 44%, specificity: 73%), 19.9 mg/dl (sensitivity: 85%, specificity: 75%) and 2.53 (sensitivity: 82%, specificity: 79%), respectively.

**Conclusions:**

This study revealed a strong association between surrogates for serum lipid profile including non-HDL-C, TG/HDL-C and Diff-C and pediatric MetS. Our findings suggest that age- and gender-specific reference values of these markers were appropriate for both risk classification and long-term control of cardiovascular events in clinical assessments.

## Background

The childhood obesity epidemic has increased dramatically over the past decades in parallel with an increase in prevalence of metabolic syndrome (MetS) in children and adolescents [1, 2]. In Iran during the past decades, transition towards a sedentary lifestyle and unhealthy dietary pattern, has resulted in higher prevalence of MetS and its complications, especially among children and adolescents [3–5]. MetS in childhood can promote the risk of chronic diseases including diabetes mellitus, atherosclerosis and cardiovascular diseases (CVD), so early diagnosis and age-specific interventions have a substantial benefit in the prevention of its complications in adulthood [6].

Although commonly measured lipid parameters such as triglyceride (TG), high-density lipoprotein cholesterol (HDL-C) and low-density lipoprotein cholesterol (LDL-C) can be acceptable alternatives for identifying individuals with CVD risks, recent studies show that non-conventional lipid profiles including non-high-density lipoprotein cholesterol (non-HDL-C), TG to HDL-C ratio (TG/HDL-C) or difference between non-HDL-C and LDL-C (Diff-C) are better than individual lipids in predicting cardiovascular events [7–9].

Non-HDL-C is an indicator of dyslipidemia calculated by subtracting high-density lipoprotein (HDL) cholesterol from total cholesterol (TC) and reflects the cholesterol in all atherogenic lipoprotein particles [10, 11]. The Adult Treatment Panel III of the National Cholesterol Education Program has considered non HDL-C as a recommended screening algorithm and American Diabetes Association and American College of Cardiology Foundation, suggested non-HDL-C as a better marker than LDL-C for predicting dyslipidemia and high-risk patients with CVD [12, 13]). Also in children, non-HDL-C has been shown to be a better indicator in anticipating dyslipidemia and subclinical atherosclerosis in adulthood compared to other lipid measures such as LDL-C [14, 15]. Because metabolic abnormalities are strongly associated with atherosclerosis, non-HDL-C can be an appropriate index for identifying individuals with MetS [16]). The strong association between non-HDL-C and MetS has been previously demonstrated [17–19]. However, the other evidences show that non-HDL-C levels varied in terms of sex, age group, and ethnic group [20]. Therefore, with respect to racial and genetic heterogeneities within and among populations, it seems necessary to determine the specific cut-points of non-HDL-C for each population. Thus, this study aims at evaluating the association between non-HDL cholesterol and MetS among Iranian children and adolescents and determining the optimal cut-off points of non-HDL-C fractions for recognition of MetS in these age groups. This study also determined the optimal cut-off points of TG/HDL-C ratio and Diff-C as important surrogate markers in cardiovascular risks.

## Methods

### Study design and population

The present study was conducted in the framework of the fifth survey of a national school-based surveillance survey entitled “Childhood and Adolescence Surveillance and Prevention of Adult Non-communicable Disease” (CASPIAN V) in 2015. Overall, 14,400 individuals without any history of chronic diseases aged 7–18 years participated in the survey. Sampling was conducted by multistage, stratified cluster sampling method from urban and rural areas of different cities in 30 provinces of Iran. Some students were randomly selected for biochemical test and fasting blood sample was collected. Protocol of this study has been explained in details previously [21].

The study was approved by the Research and Ethics Council of Isfahan University of Medical Sciences (code: 194049). After explanation of the study objectives and protocols, both written and verbal informed consent were obtained from all parents and pediatrics, respectively.

### Anthropometric and laboratory measurements

Anthropometric measurements were performed by trained employee using calibrated instruments. Weight was measured to the nearest 0.1 kg while the subjects were minimally clothed without shoes. Height was measured in the standing position to the nearest 0.5 cm without shoes [22]. Body mass index (BMI) was calculated as weight (kg) divided by square of height (m^2^). Waist circumference (WC) was measured by a non-elastic tape to the nearest 0.1 cm. Two measurements of blood pressure (BP) were done in the sitting position after 15 min of rest on the right arm using a standardized mercury sphygmomanometer. The first and fifth Korotkoff sounds were recorded as systolic blood pressure (SBP) and diastolic blood pressure (DBP), respectively.

Fasting blood samples were obtained from participants after a 12–14 h overnight fast. Fasting blood glucose (FBG), total cholesterol (TC), low density lipoprotein-cholesterol (LDL-C), high density lipoprotein-cholesterol (HDL-C), and triglycerides (TG) were measured enzymatically by Hitachi auto-analyzer (Tokyo, Japan). Non-HDL-C concentration was calculated by subtracting HDL-C concentration from total cholesterol concentration. Diff-C was defined as the difference between non-HDL-Cholesterol and LDL-Cholesterol. Atherogenic index was defined as the ratio between TG and HDL-C concentrations (TG/HDL-C) (19).

### Definition

According to the modified Adult Treatment Panel III (ATP III) criteria modified for the pediatric age group, the diagnosis of MetS was established when three or more of the following components are present:

TG concentration of 150 mg/dl or greater; HDL-C concentration of 40 mg/dL or less; FBG concentration of 100 mg/dl or greater; abdominal obesity: waist to height ratio > 0.5; and either SBP or DBP greater than the 90th percentile for age, sex, and height [23].

Overweight and obesity among children were considered as a BMI between 85th and 95th percentile and BMI greater than 95th percentile for age and sex according to WHO criteria, respectively. High LDL was defined as LDL > 110 mg/dl and High TC was defined as TC > 200 mg/dl; Low HDL-C was defined as HDL < 40 mg/dl except in boys 15–19 years old, that the cut-off was < 45 mg/dl; elevated TG was defined as TG > 100 mg/dl.

### Statistical analysis

Analyses were conducted using STATA version 11.0 (STATA Statistical Software: Release 11. STATA Corp LP. Package, College Station, TX, USA(. All variables were checked for normality and presented as the mean ± standard deviation or number (percentage). The independent sample t-test was used to compare continuous variables and the Chi-square test was employed to compare proportions based on sex. Linear regression models were used to evaluate the association between non-HDL fractions and anthropometric and biochemical variables adjusting for potential confounders. Multivariate logistic regression analysis controlling for potential confounders including age, sex, living area, screen time, SES, physical activity and BMI was also performed to examine the association between non-HDL fractions and children with MetS and CVD risk factors. Data are presented as odds ratio (OR) with 95% confidence interval (CI).

The receiver operating characteristic (ROC) curve analysis was performed to determine the optimal cut-points of non-HDL fractions for the prediction of MetS (along with corresponding sensitivities and specificities). Data were also analyzed separately for different sex and age groups. The cut-off values were estimated by using the minimum value representing the maximum sum of sensitivity and specificity. The area under curve (AUC) shows the ability of non-HDL-C fraction of cut-off points to discriminate students with and without MetS. *P*-values less than 0.05 were considered statistically significant.

## Results

The study participants consisted of 3843 students (52.3% boys). General clinical and biochemical characteristics of the children and adolescents by gender are presented in Table 1. There were significant differences in mean age, waist, SBP, DBP, FBS, TC, LDL-C, and Non-HDL-C between girls and boys (*P* < 0.05). Boys had greater proportions of low HDL-C than girls (32.7% VS 26.0%, *P* < 0.001). However, the prevalence of MetS and its other components was not significantly different between boys and girls.

**Table 1:** Characteristics of the study population: the CASPIAN-V study

Variable	Boy	Girl	Total	*P*-value
Age (year)^a^	12.39 (3.1)	12.17 (3.1)	12.28 (3.1)	< 0.001
BMI (kg/m2)^a^	18.48 (4.9)	18.53 (4.4)	18.51 (4.7)	0.565
Waist (cm)^a^	67.65 (12.8)	65.76 (11.3)	66.72 (1.2)	< 0.001
SBP (mmHg) ^a^	99.55 (13.4)	98.77 (12.8)	99.17 (13.0)	< 0.001
DBP (mmHg) ^a^	64.08 (10.7)	63.57 (10.1)	63.83 (10.4)	0.004
TG (mg/dl)^a^	87.15 (45.5)	89.02 (44.7)	88.04 (4.5)	0.201
HDL-C (mg/dl)^a^	46.21 (10.1)	46.15 (9.7)	46.18 (9.9)	0.863
LDL-C (mg/dl)^a^	89.31 (22.9)	90.86 (22.2)	90.05 (22.6)	0.034
TC (mg/dl)^a^	152.95 (28.0)	154.82 (26.6)	153.84 (27.4)	0.035
FBS (mg/dl)^a^	92.06 (12.9)	91.19 (11.1)	91.64 (12.1)	0.027
Non-HDL-C (mg/dl)^a^	106.74 (25.8)	108.66 (24.4)	107.66 (25.1)	0.018
Diff-Chol (mg/dl)^a^	17.43 (9.1)	17.80 (8.9)	17.60 (9.0)	0.201
TG/HDL^a^	2.03 (1.3)	2.08 (1.3)	2.05 (1.3)	0.265
MetS^b^	108 (5.5)	80 (4.5)	188 (5.0)	0.174
Low HDL^b^	658 (32.7)	476 (26.0)	1134 (29.5)	< 0.001
High LDL^b^	341 (16.9)	333 (18.2)	674 (17.5)	0.310
High TC^b^	100 (5.0)	89 (4.9)	189 (4.9)	0.878
Abdominal obesity^b^	1550 (21.6)	1422 (20.5)	2972 (21.1)	0.087
High FBS^b^	96 (4.8)	65 (3.8)	161 (4.2)	0.060
High TG^b^	541 (26.9)	524 (28.6)	1065 (27.7)	0.228
High SBP^b^	210 (3.0)	228 (3.3)	438 (3.1)	0.255
High DBP^b^	746 (10.5)	704 (10.2)	1450 (10.4)	0.510
High BP^b^	815 (11.5)	789 (11.4)	1604 (11.5)	0.45

^a^Data are presented as mean (SD)

^b^Data are presented as number (percentage)

*SBP* systolic blood pressure, *DBP* Diastolic blood pressure, *BP* blood pressure, *TG* triglycerides, *FBG* fasting blood glucose, *HDL-C* high-density lipoprotein-cholesterol, *TC* total cholesterol, *LDL-C* low-density lipoprotein cholesterol

low HDL: < 40 mg/dL (except in boys 15–19 y old, that cut-off was < 45 mg/dL); high LDL: > 110 mg/dL; high TG: 100 mg/dL; high TC: > 200 mg/dL; elevated FBS > 100 mg/dL; high blood pressure: > 90th (adjusted by age, sex, height)

The results of linear regression models between non-HDL-C fractions and anthropometric and biochemical variables are shown in Table 2. Non-HDL-C and TG/HDL-C were positively associated with TG in three models (*P* < 0.05). HDL-C was negatively associated with Diff-C and TG/HDL-C (*P* < 0.05). LDL-C was positively associated with Non-HDL-C and Diff-C (*P* < 0.05). Moreover, TC and FBS were positively associated with Non-HDL-C, Diff-C and TG/HDL-C in all regression models (*P* < 0.05). Table 3 presents the odds ratios and 95% confidence interval of obesity and MetS and its components associated with non-HDL-C fractions, using three logistic regression models. The odds of high TC, high LDL-C, MetS and low HDL-C were increased in pediatrics with higher non-HDL-C, Diff-C and TG/HDL-C (*P* < 0.05). Theses associations remained significant after adjusting for age, sex, living area, socio-economic status, screen time, physical activity and BMI (*P* < 0.05). There were no statistically significant associations between obesity, abdominal obesity and high blood pressure and non-HDL-C fractions. On the other hand, high FBS was significantly associated with Diff-C and TG/HDL-C (OR: 1.03, 95% CI: 1.02–1.05 and OR: 1.28, 95% CI: 1.17–1.40, respectively). Furthermore, MetS was significantly associated with non- HDL-C, Diff-C and TG/HDL-C (OR: 1.01, 95% CI: 1.01–1.02, OR: 1.08, 95% CI: 1.07–1.10 and OR: 1.9, 95% CI: 1.8–2.19, respectively).

**Table 2:** Associations between atherogenic indices and metabolic characteristics: the CASPIAN-V study

Variable	Model	ZBMI	WC	SBP	DBP	TG	HDL-C	LDL-C	TC	FBS
		B (SE)	B (SE)	B (SE)	B (SE)	B (SE)	B (SE)	B (SE)	B (SE)	B (SE)
Non-HDL-C (mg/dl)	Model I	0.001 (0.00)	0.001 (0.00)	0.001(0.00)	0.00(0.00)	0.80(0.02)^*^	0.01 (0.00)^*^	0.83(0.00)^*^	1.01(0.00)^*^	0.02(0.00)^*^
	Model II	0.001(0.00)	0.01 (0.00)	0.01(0.00)	0.00 (0.00)	0.79 (0.02)^*^	0.01 (0.00)	0.84(0.00)^*^	1.01(0.00)^*^	0.02(0.00)^*^
	Model III	–	–	0.00(0.00)	0.00(0.00)	0.78(0.02)^*^	0.01 (0.00)	0.84(0.00)^*^	1.01(0.00)^*^	0.02(0.00)^*^
Diff-C (mg/dl)	Model I	0.001(0.00)^*^	0.03 (0.02)	0.03(0.02)	0.02(0.01)	0.23(0.04)^*^	−0.28(0.01)^*^	0.25(0.04)^*^	0.96(0.04)^*^	0.22(0.02)^*^
	Model II	0.001 (0.00)^*^	0.04(0.02)	0.03(0.02)	0.02(0.01)	0.20(0.03)^*^	−0.30(0.01)^*^	0.29(0.04)^*^	0.98(0.05)^*^	0.22(0.02)^*^
	Model III	–	–	0.01(0.02)	0.01(0.01)	0.19(0.02)^*^	−0.29(0.01)^*^	0.29(0.04)^*^	0.99(0.05)^*^	0.22(0.02)^*^
Atherogenic index	Model I	0.04(0.01)^*^	0.28(0.14)	0.17(0.15)	0.14(0.12)	30.92(0.22)^*^	−3.88(0.10)^*^	0.10(0.27)	2.40(0.32)^*^	1.30(0.14)^*^
	Model II	0.03(0.01)^*^	0.26(0.13)	0.11(0.15)	0.14(0.12)	30.60(0.24)^*^	−3.99(0.11)^*^	0.26(0.29)	2.39(0.35)^*^	1.30(0.15)^*^
	Model III	–	–	0.00(0.15)	0.06(0.12)	30.57(0.24)^*^	−3.98(0.11)^*^	0.26(0.29)	2.39(0.35)^*^	1.31(0.15)^*^

^*^ Statistically significant, *SBP* systolic blood pressure, *DBP* diastolic blood pressure, *BP* blood pressure, *TG* triglycerides, *FBG* fasting blood glucose, *HDL-C* high-density lipoprotein-cholesterol, *TC* total cholesterol, *LDL-C* low-density lipoprotein cholesterol, *WC* waist circumference, *zBMI* z score of body mass index

Non–HDL-C concentration was calculated by subtracting HDL-C value from total cholesterol concentration

Diff-C was defined as the difference between total amount of non-HDL cholesterol and LDL-C (Diff-C = [non-HDL-C]-[LDL-C]). Atherogenic index as the ratio between TG and HDL-C concentrations

Model I: crude model

Model II: adjusted for age, sex, living area, screen time, SES and physical activity

Model III: additionally adjusted for BMI except for zBMI and WC

**Table 3:** Odds ratio and 95% confidence interval for cardiometabolic risk factors: the CASPIAN-V study

Variable	Model	High TC	High LDL	MetS	Low HDL	Overweight	Abdominal obesity	Obesity	High FBS	High TG	High BP
		OR (95%CI)	OR (95%CI)	OR (95%CI)	OR (95%CI)	OR (95%CI)	OR (95%CI)	OR (95%CI)	OR (95%CI)	OR (95%CI)	OR (95%CI)
Non-HDL-C (mg/dl)	Model I	1.19 (1.17,1.22)*	1.19 (1.17,1.20)*	1.01 (1.00,1.01)*	0.99 (0.99,0.99)*	1.00 (0.99,1.00)	1.00 (0.99,1.00)	1.00 (0.99,1.00)	1.00 (1.00,1.01)	1.03 (1.03,1.03)*	0.99 (0.99,1.00)
	Model II	1.19 (1.16,1.22)*	1.19 (1.17,1.21)*	1.01 (1.00, 1.01)*	0.99 (0.99,0.99)*	1.00 (0.99,1.00)	1.00 (0.99,1.00)	1.00 (0.99,1.00)	1.00 (.99,1.01)	1.03 (1.02,1.03)*	0.99 (0.99,1.00)
	Model III	1.19 (1.16,1.22)*	1.19 (1.17,1.21)*	1.01 (1.00, 1.01)*	0.99 (0.99,0.99)*	–	–	–	1.00 (.99,1.01)	1.03 (1.02,1.03)*	0.99 (0.99,1.01)
Diff-C (mg/dl)	Model I	1.07 (1.06,1.08)*	1.02 (1.01,1.02)*	1.07 (1.06,1.09)*	1.04 (1.03,1.05)*	1.01 (1.00,1.02)*	1.00 (0.99,1.01)	1.00 (0.99,1.01)	1.04 (1.02,1.05)*	1.02 (1.01,1.02)*	1.00 (0.98,1.01)
	Model II	1.07 (1.06,1.09)*	1.02 (1.01,1.03)*	1.08 (1.07,1.01)*	1.04 (1.04,1.05)*	1.01 (1.00,1.02)*	1.00 (0.99,1.01)	1.00 (0.99,1.01)	1.03 (1.02,1.05)*	1.03 (1.02,1.04)*	1.00 (0.99,1.01)
	Model III	1.07 (1.06,1.09)*	1.02 (1.01,1.03)*	1.08 (1.07, 1.10)*	1.04 (1.04,1.05)*	–	–	–	1.03 (1.02,1.05)*	1.02 (1.01,1.03)*	1.00 (0.98,1.01)
Atherogenic index	Model I	1.36 (1.26,1.46)*	1.02 (0.96,1.08)	1.8 (1.7, 2.00)*	2.43 (2.25,2.63)*	1.08 (1.00,1.16)*	1.00 (0.95,1.06)	1.03 (0.95,1.10)	1.29 (1.19,1.40)*	38.69 (29.85,50.14)*	1.00 (0.92,1.08)
	Model II	1.34 (1.24,1.46)*	1.03 (0.96,1.10)	1.9 (1.7, 2.14)*	2.51 (2.30,2.73)*	1.07 (0.98,1.15)	1.01 (0.95,1.08)	1.03 (0.95,1.12)	1.29 (1.18,1.41)*	40.33 (30.45,53.43)*	1.02 (0.94,1.11)
	Model III	1.35 (1.24,1.47)*	1.03 (0.96,1.10)	1.9 (1.8, 2.19)*	2.50 (2.30,2.72)*	–	–	–	1.28 (1.17,1.40)*	40.26 (30.36,53.40)*	1.00 (0.92,1.09)

* Statistically significant, BP blood pressure, *TG* triglycerides, *FBG* fasting blood glucose, *HDL-C* high-density lipoprotein-cholesterol, *TC* total cholesterol, *LDL-C* low-density lipoprotein cholesterol;

overweight: BMI; 85th–95th; obesity, BMI > 95th; low HDL: < 40 mg/dL (except in boys 15–19 y old, that cut-off was < 45 mg/dL); high LDL: > 110 mg/dL; high TG: 100 mg/dL; high TC: > 200 mg/dL; elevated FBS > 100 mg/dL; high blood pressure: > 90th (adjusted by age, sex, height); Non–HDL-C concentration was calculated by subtracting HDL-C value from total cholesterol concentration. Diff-C was defined as the difference between total amount of non-HDL cholesterol and LDL-C (Diff-C = [non-HDL-C]-[LDL-C]). Atherogenic index as the ratio between TG and HDL-C concentrations

Model 1: crude model

Model 2: adjusted for age, sex, living area, screen time, SES and physical activity

Model3: additionally adjusted for BMI except for overweight, obesity and abdominal obesity

Results of the ROC analyses for gender and age groups are presented in Table 4. Non-HDL-C values for predicting MetS in boys, girls and the total pediatrics were considered to be 119.5 (sensitivity: 49%, specificity: 73% and AUC: 61%), 115.5 (sensitivity: 49%, specificity: 64% and AUC: 56%) and 120.5 (sensitivity: 44%, specificity: 73% and AUC: 58%), respectively. The Diff-C thresholds of 19.9 predicted MetS in boys (sensitivity: 84%, specificity: 76% and AUC: 80%), girls (sensitivity: 86%, specificity: 74% and AUC: 80%) and the total pediatrics (sensitivity: 85%, specificity: 75% and AUC: 80%). TG/HDL-C thresholds of 2.53 (sensitivity: 80%, specificity: 80% and AUC: 80%), 2.54 (sensitivity: 86%, specificity: 79% and AUC: 83%) and 2.53 (sensitivity: 82%, specificity: 79% and AUC: 81%) were considered in order to predict MetS in boys, girls and the total pediatrics, respectively.

**Table 4:** Receiver operator curve for Non–High-Density lipoprotein fractions for identifying children with MetS

Variable	Age-sex group	Cut-off points (95% CI)	Sensitivity (95% CI)	Specificity (95% CI)	AUC
Non-HDL-C (mg/dl)	7–10 y				
	Boy	163 (96.29, 227.70)	0.69 (0.585, 0.807)	0.73 (0.48, 0.97)	0.71
	Girl	104.5 (88.21, 119.78)	0.71 (0.47, 0.95)	0.50 (0.28, 0.70)	0.60
	Total	104.5 (73.99, 134.00)	0.60 (0.28, 0.91)	0.50 (0.14, 0.85)	0.55
	11–14 y				
	Boy	119.5 (107.00,130.99)	0.54 (0.34,0.74)	0.73 (0.59,0.85)	0.64
	Girl	116.5 (77.57,154.42)	0.49 (0.10, 0.86)	0.65 (0.21, 1.07)	0.57
	Total	116.5 (111.38, 120.61)	0.53 (0.37, 0.68)	0.67 (0.57, 0.76)	0.60
	15–18 y				
	Boy	101.5 (81.67,120.32)	0.91 (0.66,1.16)	0.51 (0.27,0.73)	0.71
	Girl	115.5 (75.49, 154.50)	0.54 (0.07, 1.00)	0.64 (0.14, 1.14)	0.59
	Total	104.5 (83.66, 124.33)	0.79 (0.52, 1.06)	0.52 (0.25, 0.78)	0.65
	7-18y				
	Boy	119.5 (103.37, 134.62)	0.49 (0.26, 0.71)	0.73 (0.50, 0.95)	0.61
	Girl	115.5 (88.58, 141.41)	0.49 (0.18, 0.78)	0.64 (0.25, 1.01)	0.56
	Total	120.5 (105.33, 134.66)	0.44 (0.22, 0.65)	0.73 (0.52, 0.93)	0.58
Diff-C (mg/dl)	7–10 y				
	Boy	19.9 (17.47, 22.12)	0.78 (0.60, 0.95)	0.78 (0.72, 0.83)	0.78
	Girl	20.7 (15.94, 25.25)	0.89 (0.75, 1.00)	0.78 (0.66, 0.89)	0.84
	Total	19.9 (18.88,20.71)	0.84 (0.73,0.93)	0.77 (0.72,0.81)	0.80
	11–14 y				
	Boy	19.9 (18.26, 21.33)	0.83 (0.71, 0.93)	0.77 (0.72, 0.81)	0.80
	Girl	19.9 (18.18, 21.41)	0.82 (0.69, 0.94)	0.73 (0.64, 0.82)	0.78
	Total	19.9 (18.41, 21.18)	0.82 (0.74,0.90)	0.75 (0.71,0.78)	0.79
	15–18 y				
	Boy	19.9 (18.05, 21.54)	0.91 (0.83, 0.99)	0.74 (0.67, 0.80)	0.83
	Girl	20.5 (15.45, 25.34)	0.92 (0.75, 1.08)	0.76 (0.68, 0.84)	0.84
	Total	19.9 (19.16,20.43)	0.92 (0.84,0.99)	0.74 (0.70,0.77)	0.83
	7–18 y				
	Boy	19.9 (19.26,20.33)	0.84 (0.76,0.91)	0.76 (0.73,0.79)	0.80
	Girl	19.9 (19.37,20.22)	0.86 (0.78,0.93)	0.74 (0.70,0.78)	0.80
	Total	19.9 (19.76, 19.83)	0.85 (0.79,0.90)	0.75 (0.74,0.76)	0.80
Atherogenic index	7–10 y				
	Boy	2.53 (1.83,3.23)	0.70 (0.52,0.88)	0.83 (0.70,0.95)	0.77
	Girl	2.57 (2.03,3.10)	0.93 (0.83,1.02)	0.81 (0.69,0.92)	0.87
	Total	2.53 (2.10,2.96)	0.82 (0.72,0.90)	0.81 (0.71,0.91)	0.82
	11–14 y				
	Boy	2.58 (2.37,2.78)	0.80 (0.70,0.90)	0.82 (0.78,0.86)	0.81
	Girl	2.54 (1.96,3.11)	0.79 (0.65,0.93)	0.77 (0.61,0.93)	0.78
	Total	2.54 (2.25,2.83)	0.80 (0.70,0.89)	0.80 (0.72,0.87)	0.80
	15–18 y				
	Boy	2.48 (2.04,2.91)	0.89 (0.75,1.01)	0.75 (0.65,0.83)	0.82
	Girl	2.73 (2.24,3.22)	0.92 (0.79,1.05)	0.82 (0.75,0.88)	0.87
	Total	2.48 (2.17,2.78)	0.90 (0.79,.99)	0.76 (0.69,0.82)	0.83
	7–18 y				
	Boy	2.53 (2.35,2.71)	0.80 (0.71,0.88)	0.80 (0.76,0.83)	0.80
	Girl	2.54 (2.19,2.89)	0.86 (0.77,0.94)	0.79 (0.71,0.86)	0.83
	Total	2.53 (2.41,2.65)	0.82 (0.76,0.88)	0.79 (0.76,0.82)	0.81

*CI* confidence interval, *AUC* area under curve, shown as percentage

Non–HDL-C concentration was calculated by subtracting HDL-C value from total cholesterol concentration. Diff-C was defined as the difference between total amount of non-HDL cholesterol and LDL-C (Diff-C = [non-HDL-C]-[LDL-C]). Atherogenic index as the ratio between TG and HDL-C concentrations

## Discussion

In the current study, we presented the age- and sex-stratified cut off values of serum lipid profiles including non-HDL-C, TG/HDL-C and Diff-C for predicting MetS in Iranian children and adolescents from a national school-based surveillance survey. The thresholds were determined 120 mg/dl, 2.53 and 19.9 mg/dl for non-HDL-C, TG/HDL-C ratio and Diff-C, respectively in the total participants. The findings demonstrated that the higher values of non-HDL-C, TG/HDL-C and Diff-C were associated with increased risk of MetS in this age group.

Several studies have recommended the use of non- HDL-C as a valuable predictor for dyslipidemia and cardiovascular risks [19, 24, 25]. Because dyslipidemia is a hallmark of metabolic syndrome and prevalence of dyslipidemia in Iran is high [26–28] , this study evaluated the relation between non-HDL-C and MetS. The reference values for non-conventional lipid profiles such as non-HDL-C for recognition of MetS in children and adolescents have been determined in few studies. In a survey on US children and adolescents aged 12–19 years, non-HDL-C was strongly associated with MetS and its thresholds was determined 120 mg/dl and 145 mg/dl to indicate borderline and high MetS risk, respectively [29]. Consistent with US study, this study showed that non-HDL-C above 120 mg/dl was associated with MetS in Iranian children and adolescents. The Expert Panel on Integrated Guidelines for Cardiovascular Health and Risk Reduction in Children and Adolescents has suggested the use of the non-fasting non-HDL-C level and non-HDL-C ≥ 145 mg/dl as a universal screening level for dyslipidemia in children and adolescents up to 19 years [30]. Shim et al. presented the age and gender-specific reference values for non-HDL-C and the TG/ HDL-C in Korean children and adolescents aged 10–19 years as a valuable information for individualized interpretations of lipid profiles and interventions as well as for strategies to prevent and manage childhood and adolescent dyslipidemia [31]. Saito et al. in a study on Japanese obese boys aged 12.0 ± 2.6 years with MetS demonstrated that higher non-HDL-C levels is associated with increased risk for the development of atherosclerosis [32].

Few studies have reported reference values for TG/HDL-C ratio and Diff-C in children and adolescents. In confirming our findings, the TG/HDL-C ratio was shown as an independent predictor of atherosclerosis in survey conducted on US adolescents and young adults [33]. In a large Italian study, the TG/HDL-C ratio is presented as a simple and effective method for diagnosis CVD and dyslipidemia in children and adolescents [34]. Even it was documented that Diff-C fraction and TG/HDL-C ratio are able to predict MetS more accurately than total non-HDL-C [19, 34] and the TG/HDL-C ratio proved a better index than HOMA-IR in screening for MetS in obese children and adolescents [35]. However, non-HDL-C is more popular than TG/HDL-C ratio and Diff-C worldwide [36]. Overall, the consistent findings regarding the association between these lipid profile surrogates and cardiovascular risk in different population with different ethnicity, culture, food pattern and lifestyle, reveals the utility and importance of these indices in the managements of MetS in children and adolescents across the globe.

Non–HDL-C is suggested as an appropriate method for screening dyslipidemia and cardiovascular risk in the pediatric setting because overnight fasting is not necessary before the measurement of non–HDL-C [30]. Furthermore, since TG variability can lead to significant changes in LDL-C evaluation in children and adolescents, non–HDL-C level can be more reliable than LDL-C concentration in this age group [37]. In fact, Non-HDL-C reflects the amounts of the TG-rich lipoprotein content of several atherogenic particles, including LDL-C, intermediate-density lipoprotein cholesterol (IDL-C), very–low-density lipoprotein cholesterol (VLDL-C) and chylomicron remnants. It has been indicated that IDL and small VLDL values, but not LDL value, associate with increased risk of atherosclerosis [38, 39]. The atherogenic effect of small TG-rich lipoproteins is due to their association with clotting and the fibrinolytic pathway through producing foam cells [40]. Moreover, non-HDL-C indicates an estimate of all ApoB-containing lipoproteins, all of which have the potential to take cholesterol into the arterial wall and result in atherosclerotic lesions. Therefore, non-HDL-C can mirrors atherogenic risk not captured by LDL-C measurement alone, especially in the case of elevated TG [41].

In this study, among five components of MetS, high TG and low HDL-C were significantly associated with non–HDL-C. Non–HDL-C contains atherogenic lipoproteins, including LDL-C, IDL-C, VLDL-C and correlates highly with serum TG levels. Because serum TG is mainly carried in lipid-rich particles including VLDL, fasting serum TG levels strongly associates with VLDL-C concentration. Besides, high serum TG levels often are accompanied by abnormal levels of small particles of LDL and low HDL-C concentration [42]. However, TG and non–HDL-C have extremely different biochemical specifications and metabolic mechanisms; non–HDL-C mostly via atherogenic process, and TG mostly via insulin resistance, are associated with CVD risk [43].

For the first time, this study presents a sex and age-stratified cut-off values of non-HDL-C, TG/HDL-C and Diff-C for predicting MetS in a nationally representative sample of Iranian children and adolescents aged 7–18 years and evaluates the association between non–HDL-C concentration and MetS in multivariable logistic regression analyses based on standard protocols. Nevertheless, this study has some limitations which have to be pointed out. First, the cross-sectional design of this study did not let us to obtain any causality between non–HDL-C concentration and MetS. Second, we had no data regarding the prevalence of atherosclerosis or CVD in these children and adolescents so we could not show inferences on the association between non–HDL-C and these events as definite end points. Further studies are suggested to assess the association between lipid profile surrogates in children and adolescents and cardiovascular status of parents. Moreover, performing specific cardiac physical examinations including ecocolordoppler with intima- media thickness (IMT) measurement could provide more accurate information to clarify these associations.

## Conclusion

According to racial and genetic heterogeneities between populations, we determined the specific cut-points of serum lipid profiles including non-HDL-C, TG/HDL-C ratio and Diff-C for predicting MetS in 7–18 year-olds Iranian children and adolescents. The robust association between these lipid profile surrogates and both current MetS and future atherosclerosis supported the use of these parameters as appropriate methods in both risk classification and long-term control of CVD risk in the clinical assessments.

## Abbreviations

ATP III: Adult treatment panel III
BMI: Body mass index
BP: Blood pressure
CASPIAN: Childhood and adolescence surveillance and prevention of adult non-communicable disease
CI: Confidence interval
CVD: Cardiovascular diseases
DBP: Diastolic blood pressure
Diff-C: Difference between non-high-density lipoprotein cholesterol and low-density lipoprotein cholesterol
FBG: Fasting blood glucose
HDL-C: High-density lipoprotein cholesterol
LDL-C: Low-density lipoprotein cholesterol
MetS: Metabolic syndrome
Non-HDL-C: Non-high-density lipoprotein cholesterol
OR: Odds ratio
ROC: Receiver operating characteristic
SBP: Systolic blood pressure
SD: Standard deviation
TG: Triglycerides
TG/HDL-C: Triglycerides to high-density lipoprotein cholesterol ratio
WC: Waist circumference
